# Homology modelling of metabotropic glutamate receptor 2

**DOI:** 10.1186/1758-2946-3-S1-P40

**Published:** 2011-04-19

**Authors:** S Mordalski, T Kosciolek, A Ravna, I Sylte, AJ Bojarski

**Affiliations:** 1 Department of Medicinal Chemistry, Institute of Pharmacology Polish Academy of Sciences, Krakow, 31-343, Poland; 2 Medical Pharmacology and Toxicology, Department of Medical Biology, Faculty of Health Science, University of Tromsø, N-9037 Tromsø, Norway

## 

Many studies show involvement of metabotropic glutamate receptors (mGluRs) in synaptic excitation transudation. The mGluR family consists of eight proteins divided into three groups corresponding to sequence similarities, pharmacology and physiological role. These groups are: I (mGluR1, -5), II (mGluR2, -3) and III (mGluR4, -6, -7, -8). Group II lies in field of our interest due to its potential as therapeutic target for stroke and pain drugs. Primary goal of this research is to create viable virtual model of transmembrane domain of mGluR2 receptor capable of binding reference ligands. This model will be used for further research.

Our approach is based on homology modeling. Rhodopsin crystal structure has been used as a template for creating mGluR2 models. Due to inconsistencies between sequence alignments found in literature our alignment has been prepared basing on experimental secondary structure prediction for mGluR1 and mGluR3 [1]. So created models were then tested against available mutational data [2,3] and by flexible docking of known active/inactive compounds.
